# Exploring perceptions, readiness, barriers, and facilitators related to the potential implementation of postpartum depression screening: A mixed-methods study in a Lebanese maternity setting

**DOI:** 10.1371/journal.pone.0354470

**Published:** 2026-07-30

**Authors:** Lama El Haj Sleiman, Linda Abou-Abbas

**Affiliations:** 1 International Committee of the Red Cross, Beirut, Lebanon; 2 ESA business school (Ecole Supérieure des Affaires), Beirut, Lebanon; 3 INSPECT-LB (Institut National de Santé Publique Epidémiologie Clinique Et Toxicologie-Liban), Beirut, Lebanon; Jawaharlal Institute of Postgraduate Medical Education and Research, INDIA

## Abstract

**Background:**

Postpartum depression (PPD) is a common mental health condition affecting mothers, infants, and families, with higher prevalence in middle-income countries. Despite its burden, PPD remains under-screened in Lebanese public hospitals, particularly in disadvantaged areas like Tripoli.

**Objectives:**

To assess nurses’ knowledge, attitudes, and practices regarding PPD and explore facilitators, and barriers influencing the potential implementation of systematic PPD screening.

**Methods:**

A mixed-methods study was conducted in a Lebanese public university hospital, combining a quantitative survey with qualitative focus group discussions involving nurses and managers. Descriptive analyses were applied to survey data, and thematic analysis was used for qualitative findings.

**Results:**

Most nurses were aware of PPD (92.9%) and “baby blues” (92.9%), but only 21.4% were aware of standardized screening tools. Correct identification of key symptoms was reported by 71.4%, while only 35.7% correctly identified all risk factors and 28.6% recognized appropriate combined management (psychotherapy and antidepressants). Regarding attitudes, all participants (100%) agreed that PPD screening should be part of routine postnatal care, and 92.9% recognized its seriousness and the role of nurses in early detection. However, only 78.6% felt confident identifying PPD signs. In terms of practice, only 28.6% consistently assessed mothers’ emotional well-being before discharge. Qualitative findings suggested a generally positive attitude toward the potential implementation of screening, while also highlighting perceived barriers, including insufficient training, unclear role delineation, inadequate referral pathways, time constraints, and weak internal communication regarding mental health services.

**Conclusion:**

The findings suggest that challenges to the potential implementation of PPD screening may be related to capacity and organizational support than to resistance among participants. Strengthening training, integrating standardized screening into routine postnatal care, clarifying roles and referral pathways may facilitate the implementation of PPD screening. Given the exploratory nature of this single-center study, further research is needed to assess the transferability of these findings to other maternity care settings in Lebanon.

## 1. Introduction

Postpartum depression (PPD) is one of the most common mental health complications associated with childbirth, affecting approximately one in five women during the perinatal period [1,2]. The postpartum period is a particularly vulnerable phase, during which women may develop depressive and anxiety disorders at higher rates than other obstetric complications. The World Health Organization (WHO) defines PPD as a common mental health condition that can emerge within weeks or months after delivery. PPD can adversely affect maternal well-being, mother–infant bonding, infant growth and development, and family functioning if left unrecognized and untreated [3].

Global prevalence estimates range from 10% to 20%, but rates are significantly higher in middle-income countries, where women may experience two to three times the risk observed in high-income settings [2,3]. In the Middle East, the prevalence reaches 27%, underscoring the substantial burden in this region [4]. PPD is multifactorial, influenced by psychosocial, biological, socioeconomic, and obstetric factors. Well-established risk factors include low education, unplanned pregnancy, low socioeconomic status, insufficient social support, intimate partner violence, young maternal age, psychiatric history, and pregnancy or neonatal complications such as NICU admission [4–6]. These risk factors often accumulate, disproportionately affecting women living in precarious contexts.

Symptoms of PPD include persistent sadness, loss of interest, sleep disturbances, feelings of guilt or worthlessness, difficulty bonding with the infant, and, in severe cases, suicidal ideation requiring urgent intervention [7]. These symptoms must be differentiated from “baby blues,” a transient and self-limited condition that resolves within two weeks [8]. The consequences of PPD extend beyond maternal well-being. It can impair mother–infant bonding, increase the risk of self-harm, and negatively influence the child’s socio-emotional development, cognitive functioning, attention, and behavioral regulation [9–12]. Evidence also suggests long-term physical and mental health risks among children exposed to maternal PPD [2,13]. Paternal depression is also more common when mothers experience PPD, reaching up to 50% in some studies, which further strains family functioning [14,15]. Due to these far-reaching consequences, PPD is recognized as a major public health concern [16].

Despite its prevalence, up to 75% of perinatal mental health conditions remain undiagnosed or untreated, largely due to lack of awareness, insufficient screening, and limited mental health infrastructure [17]. While the American College of Obstetricians and Gynecologists (ACOG) recommends screening for depression during the first postnatal visit, an estimated 40% of women do not attend this appointment, prompting recommendations for earlier screening before hospital discharge [18].

In Lebanon, PPD affects approximately 21% of postpartum women, with rates rising to nearly 60% during the COVID-19 pandemic [19,20]. Although national health strategies increasingly emphasize mental health, routine perinatal screening is not yet implemented in public hospitals [21,22]. Tripoli, a socioeconomically disadvantaged region, is heavily impacted by poverty, instability, and limited access to healthcare services. Tripoli Governmental Hospital (TGH), the second-largest public hospital in the country, serves a predominantly vulnerable population, with occupancy rates exceeding 90% and 250–300 births per month, placing considerable strain on staff and resources [23].

Women delivering at TGH often present multiple risk factors for PPD, including unplanned pregnancies, intimate partner violence, low socioeconomic status, limited education, obstetric complications, and inadequate social support [24]. Although psychological consultations are available free of charge in the hospital’s affiliated primary healthcare center, referral pathways are unclear and underutilized

Introducing a standardized screening tool is therefore essential. The Edinburgh Postnatal Depression Scale (EPDS), a widely validated 10-item self-report instrument developed by Cox et al. (1987), is endorsed by the WHO and adapted to Arabic-speaking populations [13]. Its successful use at Hôtel-Dieu de France, a private hospital in Beirut, demonstrates its feasibility prior to postpartum discharge [25]. Given its simplicity, accessibility, and cultural appropriateness, the EPDS represents a practical and evidence-based tool for use at TGH. Implementing structured PPD screening at TGH is thus a potential step toward early identification of at-risk women, timely referral to appropriate services, and improved maternal mental healthcare within this public institution. This study aimed to explore nurses’ knowledge, attitudes, and practices (KAP) regarding PPD screening, as well as perceived barriers and facilitators influencing potential implementation.

## 2. Methods

### 2.1. Study design and setting

This sequential explanatory mixed-methods study was conducted at the maternity department of TGH in northern Lebanon, from July 10 to August 10, 2025. TGH was purposefully selected because of its unique contextual characteristics, including a high obstetric workload, a predominantly socioeconomically vulnerable patient population, and limited healthcare resources, as described in the Introduction. Given the exploratory nature of the study and the absence of routine postpartum depression screening in Lebanese public hospitals, a single-setting approach was considered appropriate to generate in-depth, context-specific evidence and inform future implementation efforts in similar settings. Phase 1 (quantitative) assessed obstetric nurses’ knowledge, attitudes, and practices regarding PPD screening via questionnaire. Phase 2 (qualitative) explored perceptions, barriers, and facilitators related to the potential implementation of the EPDS in clinical practice via focus groups, informed by Phase 1 results.

### 2.2. Phase 1: Quantitative assessment of KAP

A cross-sectional study design was used to assess obstetric nurses’ KAP regarding PPD and its screening. A total population sampling approach was adopted, whereby all obstetric nurses working in the maternity department at TGH during the study period were eligible and included. Given the fixed and small workforce size (n = 14), no sampling strategy was applied.

Data were collected using a self-administered anonymous questionnaire (S1 File), adapted from previously validated instruments in the literature [26,27] and modified to suit the local hospital context. The questionnaire assessed three main domains: (1) knowledge, defined as nurses’ understanding of PPD signs, symptoms, risk factors, consequences, and management; (2) attitudes, referring to nurses’ beliefs, perceptions, and opinions regarding maternal mental health and PPD screening; and (3) practices, defined as self-reported actions related to the identification, assessment, referral, or management of women experiencing symptoms of PPD.

To ensure linguistic and cultural appropriateness, the questionnaire was translated into Arabic by a bilingual researcher with expertise in medical terminology. Face validity was assessed through independent review by two experienced midwives, who evaluated clarity, relevance, and comprehensibility. Minor revisions were incorporated based on their feedback.

No formal pilot testing or psychometric validation (e.g., construct validity or internal consistency reliability testing) was conducted, as the instrument was used for descriptive purposes within a small total population sample.

Given the exploratory nature of the study and the limited sample size, the findings are intended to describe knowledge, attitudes, and practices rather than to validate the measurement properties of the instrument.

Data were collected using Device Magic, a secure mobile data collection platform ensuring confidentiality and data protection. Eligible participants received an informed consent form, explaining the study’s purpose, voluntary nature, anonymity, and right to withdraw at any time. Upon confirmation of consent, the questionnaire link was shared individually via WhatsApp. Participants were allowed to complete the questionnaire during their shifts at their convenience to minimize disruption to clinical duties. Reminder messages were sent once after one week to non-respondents. All 14 eligible nurses completed the questionnaire, yielding a 100% response rate. Completion time averaged 10–15 minutes per participant.

Quantitative data from the questionnaire were analyzed descriptively using Microsoft Excel. Descriptive statistics, including frequencies and percentages, were calculated for all KAP variables.

### 2.3. Phase 2: Qualitative assessment: Exploration of perceptions, readiness, barriers, and facilitators for the potential implementation of the EPDS

To explore the perceptions, readiness, barriers, and facilitators of the potential implementation of the EPDS as a routine screening tool at TGH, focus group discussions (FGD) were conducted. This qualitative approach was selected to explore healthcare staff perceptions, attitudes, motivations, barriers, facilitators, and resource requirements in depth, capturing interactive dynamics and diverse viewpoints that quantitative methods alone cannot provide. The discussions were built directly on Phase 1 questionnaire findings to ensure relevance and address identified gaps.

Five focus groups were organized:

- Four groups with obstetric nurses (2–4 participants per group; 12 of 14 participated, with two absent due to maternity or annual leave).
- One group with key managerial personnel (head nurse, supervising midwife, and head of the obstetrics and gynecology department).

A semi-structured topic guide (S2 File) was developed from Phase 1 results and pilot tested. It covered PPD awareness, risk factors, perceived frequency/importance, current distress identification practices, perceptions of workload and resource requirements, staff acceptability, concerns, facilitators, patient reactions, cultural factors, training needs, barriers/facilitators, referral pathways post-positive screen and closing suggestions.

Focus groups were held in a private, confidential space within TGH between late July and early August 2025 to minimize disruptions. Sessions, lasting 45–60 minutes each, were facilitated by the principal investigator, who used open-ended prompts to encourage balanced participation. Audio recordings were obtained with explicit participant consent, and field notes captured non-verbal cues.

The qualitative data collection emphasized participant engagement and interaction, allowing participants to respond to and reflect on each other’s comments. This method enriched the data by highlighting consensus, discrepancies, and nuanced perspectives on barriers, facilitators, and resource requirements for EPDS implementation. The data collected provided a detailed and context-specific understanding of organizational, cultural, and practical factors that could affect the implementation of structured PPD screening at TGH.

Discussions were conducted in Arabic, transcribed verbatim, and de-identified to protect anonymity. A manual thematic analysis was performed following Braun and Clarke’s framework [28]. Two researchers independently reviewed and coded the transcripts using a combined inductive-deductive approach. Deductive codes were informed by the study objectives, focus group guide, and findings from Phase 1, while inductive coding allowed for the identification of unanticipated concepts emerging directly from participants’ narratives. Coding discrepancies were discussed until consensus was reached, and themes were refined through iterative comparison across transcripts.

Data collection and analysis occurred concurrently, allowing emerging findings to inform subsequent discussions. Recruitment continued until thematic saturation was achieved, defined as the point at which no substantially new themes, concepts, or insights emerged from additional focus groups. Theme consistency was assessed across participant groups to enhance credibility and confirm the robustness of interpretations.

To enhance methodological rigor, reflexivity was maintained throughout the research process. The principal investigator, who facilitated the focus groups, had a professional background in maternal health and was familiar with the clinical setting. The research team regularly discussed how their professional experiences, assumptions, and perspectives regarding maternal mental health and postpartum depression might influence data collection, coding decisions, and interpretation of findings. Reflexive discussions and analytical memos were used to critically examine potential biases and promote transparency in the analytic process.

Researcher positionality was acknowledged by recognizing that the investigators’ clinical and academic backgrounds could shape interpretation of participants’ responses. Efforts to minimize undue influence included consensus-based theme development, maintenance of an audit trail documenting coding decisions, and the use of representative participant quotations to support the credibility and trustworthiness of the findings.

### 2.4. Ethical considerations

Ethical approval for this study was obtained from the International Committee of the Red Cross (ICRC), through the Institutional Review Board CORE (LDP_CORE 25/009 CGB/nre) in July 2025. The study was conducted in accordance with the principles outlined in the Declaration of Helsinki. Written informed consent was obtained from all participants prior to their inclusion in the study. Participants were informed about the purpose of the study, the voluntary nature of participation, and their right to withdraw at any time without any consequences. Confidentiality and anonymity were strictly maintained throughout data collection, analysis, and reporting.

## 3. Results

### 3.1. Participant characteristics and response rate

All 14 obstetric nurses in TGH’s postpartum ward completed the survey (100% response rate), assessing their knowledge, attitudes, and practices regarding PPD. Regarding clinical experience, more than half of the participants (57.1%, n = 8) had more than ten years of professional practice. Three nurses (21.4%) had less than one year of experience, two (14.3%) had between one and five years, and only one nurse (7.1%) had between six and ten years of experience.

### 3.2. Quantitative analysis: KAP survey

#### 3.2.1. Knowledge of obstetric nurses regarding PPD.

Nearly all participants reported being aware of PPD (92.9%) and had heard of the term “baby blues” (92.9%). However, awareness of screening tools was low, with only 21.4% having heard of a PPD screening questionnaire. When focusing on correct knowledge, 78.6% correctly recognized that PPD is distinct from the baby blues, though only half (50.0%) correctly identified the typical duration of each. Knowledge of risk factors was limited, with only 35.7% identifying all key factors, whereas 71.4% correctly identified all major symptoms of PPD. Regarding management, 28.6% identified the combination of psychotherapy and antidepressants as an appropriate approach and 78.6% correctly identified a psychologist or psychiatrist as the most appropriate professional to manage PPD. Awareness of the consequences of untreated PPD was moderate, with 42.9% acknowledging the risk of chronic depression, suicidal ideation, and recognizing additional impacts on the child’s emotional development (Table 1).

**Table 1:** Percentage of obstetric nurses with correct knowledge regarding PPD (n = 14).

Question	Correct Answer	% Correct Answer
PPD is different from baby blues	Yes	78.6
Typical duration of baby blues vs. PPD	Baby blues ≤ 2 weeks; PPD persists beyond 2 weeks	50.0
Risk factors for PPD^1^	Unwanted or unplanned pregnancy; financial insecurity; preterm birth or infant health problems	35.7
Symptoms of PPD^1^	Sleep disturbances; persistent sadness or depressive mood; difficulty bonding with the baby	71.4
Effects of untreated PPD^1^	Chronic depression & suicidal ideation & delayed emotional development in the child	57.1
Management options for PPD^1^	Psychotherapy + antidepressants	28.6
Best person to manage PPD	Psychologist/ psychiatrist	78.6

^1^Questions allowed multiple answers (“check all that apply”), and correct percentages reflect participants who selected all correct options.

#### 3.2.2. Attitudes of obstetric nurses toward PPD screening.

Most obstetric nurses agreed that PPD is a serious condition that should be routinely screened in all postpartum women (92.9%) and 78.6% reported confidence in identifying signs of PPD in mothers. All participants (100.0%) agreed that PPD screening should be incorporated into routine postnatal care in the hospital. Additionally, 78.6% agreed that lack of time prevents healthcare professionals from adequately screening for PPD. Most nurses (92.9%) agreed that nurses and midwives play a key role in the early detection of PPD (Table 2).

**Table 2:** Attitudes of obstetric nurses toward PPD screening (n = 14).

Statement	Response	Number	Percentage (%)
**PPD is a serious condition that should be routinely screened in all postpartum women.**	**Strongly Agree/Agree**	**13**	**92.9**
	**Disagree**	**1**	**7.1**
**I feel confident identifying signs of PPD in mothers.**	**Strongly Agree/Agree**	**11**	**78.6**
	**Disagree**	**3**	**21.4**
**Screening for PPD should be part of routine postnatal care in the hospital.**	**Strongly Agree/Agree**	**14**	**100.0**
**Lack of time prevents healthcare professionals from adequately screening for PPD.**	**Strongly Agree/Agree**	**11**	**78.6**
	**Strongly disagree/Disagree**	**3**	**21.4**
**Nurses and midwives can play a key role in the early detection of PPD.**	**Strongly Agree/Agree**	**13**	**92.9**
	**Disagree**	**1**	**7.1**

#### 3.2.3. Practices of obstetric nurses in the identification and management of PPD.

Only 28.6% of participants reported consistently asking mothers about their emotional well-being before discharge, while 71.4% reported doing so only sometimes or rarely. Similarly, providing information on PPD signs before discharge was reported consistently by 21.4% of nurses, whereas 78.6% reported doing so inconsistently. Half of the participants (50.0%) consistently discussed suspected PPD cases with a doctor or nursing supervisor, while the remaining half did so inconsistently. Less than half of nurses reported having received training on mental health or PPD (42.9%), and only 35.7% indicated having sufficient knowledge and tools to support mothers emotionally (Table 3).

**Table 3:** Practices of obstetric nurses regarding PPD (n = 14).

Practice Statement	Practice Category	Number	Percentage (%)
**Asking mothers about emotional well-being before discharge**	Consistent practice (Always)	4	28.6
	Inconsistent practice (Sometimes/Rarely)	10	71.4
**Providing information on PPD signs before discharge**	Consistent practice (Always)	3	21.4
	Inconsistent practice (Sometimes/Rarely)	11	78.6
**Discussing suspected PPD cases with a doctor or nursing supervisor**	Consistent practice (Always)	7	50.0
	Inconsistent practice (Sometimes)	7	50.0
**Received training on mental health or PPD**	Consistent practice (Yes)	6	42.9
	Inconsistent practice (No)	8	57.1
**Having sufficient knowledge and tools to support mothers emotionally**	Consistent practice (Yes)	5	35.7
	Inconsistent practice (No)	9	64.3

Consistent practice was defined as responses indicating routine implementation of the practice (e.g., “always”), whereas inconsistent practice referred to practices reported as occurring only sometimes or rarely.

### 3.3. Qualitative Analysis: Exploration of perceptions, readiness, barriers, and facilitators for the potential implementation of the EPDS

Five focus groups were conducted: four with obstetric nurses (FG1, FG2, FG3, FG5) and one with managerial staff (department head, head nurse, and supervising midwife – FG4). The thematic analysis identified several major themes (S1 Table).

#### 3.3.1. Perceptions of risk factors for PPD.

Participants across all FGDs consistently identified several risk factors contributing to PPD within their context. Socioeconomic challenges were frequently mentioned, as most patients in the hospital came from disadvantaged backgrounds.

One participant in FGD2 explained,

*“At least 80% of the women who come to this hospital have a low socioeconomic status,” (FGD2, P5) while another added, “The patient population in this hospital comes from disadvantaged backgrounds, and none of them are from affluent families.”*

These financial and social hardships were seen as a significant source of stress for mothers. Cultural and familial pressures further exacerbated the risk of PPD. Participants described how societal expectations, particularly around gender preferences, played a role.

In FGD1, a participant shared, *“The husband can refuse the baby if it’s a girl and not a boy. For example, we had a case where the parents abandoned their sixth daughter at the hospital because they wanted a son.”*

Similarly, in FGD3, it was noted, *“When a woman is expecting a boy and gives birth to a girl, the whole family appears anxious, and the mother looks sad. This can cause depression.”*

Violence and abuse were also highlighted as significant contributors. In FGD1, one participant recounted, *“Today, we had a mother who gave birth to a premature baby through an emergency C-section because her husband beat her on the stomach.”*

Another participant in FGD3 described a case where *“a woman who gave birth today, and her mother-in-law beats her all the time, was threatened that she couldn’t return home with her baby.”*

Stress related to neonatal health was another recurring theme. Mothers with babies admitted to the neonatal intensive care unit (NICU) were described as particularly vulnerable to emotional distress. A participant in FGD1 explained,

“*There are many babies who need to be admitted to the NICU in our context, and this causes a lot of stress for the mothers.” Similarly, in FGD5, a participant noted, “The mothers who have babies admitted to the NICU spend their time crying. Most of the women leave the hospital crying.” (FGD5, P12)*

#### 3.3.2. Perceived prevalence of PPD.

Participants reported varying perceptions of the prevalence of PPD, with most frontline staff perceiving it as highly common in their setting. Some participants estimated extremely high prevalence, with one noting,

*“I believe 95% of our patients have postpartum depression”* (FGD2, P6),

and another stating,

*“Even 100% of our patients”* (FGD2, P6).

Others attributed the high perceived prevalence to clinical factors such as frequent NICU admissions, as expressed by one participant:

*“I think the percentage of PPD is high in our hospital because we have a large number of newborns admitted to the NICU every month” (FGD3, P7).*

In contrast, some participants highlighted challenges in identifying PPD due to short hospital stays and the absence of systematic screening. One participant explained,

*“In the few hours they spend in the hospital, we can’t recognize PPD” (FGD4, C3).*

Managers provided lower estimates, suggesting a prevalence of approximately 15–20%, which they similarly attributed to limited hospitalization duration. As one manager stated, *“I think it’s 20%” (FGD4, C1).*

#### 3.3.3. Current identification of cases.

Participants across all FGDs reported that the identification of PPD cases is currently informal, relying on observable behaviors and verbal cues from mothers. Signs such as crying, refusal to breastfeed, sadness, or anxiety often alert healthcare providers to possible emotional distress. For instance, one participant in FGD3 explained, *“We know sometimes from their expressions, their appearance, their behavior, and the way their family interacts with them.”* Similarly, in FGD5, a participant noted, *“We can tell from her face and expressions, especially if she’s not cooperating with us or seems agitated.” (FGD5, P11)*

Mothers sometimes disclose their struggles voluntarily, which helps providers identify cases. As one participant in FGD2 shared, “They talk about their worries on their own, like saying, ‘My husband left me, and I have no one,’ or ‘I don’t have money to pay for my delivery.” *(FGD2, P5)* However, participants acknowledged that many mothers do not openly express their emotions, making it difficult to identify all cases.

In FGD1, a participant observed, *“Maybe there are women who go unnoticed because we can only identify signs if we spend enough time with them, and we don’t always have that time.” (FGD1, P1)*

Participants also highlighted cultural and social barriers that prevent mothers from sharing their feelings, such as fear of judgment or violence from family members. In FGD4, a participant explained, *“The women we see are often afraid of their mothers or mothers-in-law and don’t have the courage to reveal what they’re going through.” (FGD4, C1)*

#### 3.3.4. Current management of the identified PPD cases.

In terms of managing identified cases, participants reported that they primarily offer emotional support and providing reassurance. This includes listening to mothers, understanding their concerns involving family members, and occasionally offering religious or spiritual advice.

One participant explained, “We try to communicate with her and understand her problem if she’s willing to talk. We do our best to ease her worries” (FGD5, P11). A participant in FGD1 stated, “I speak with her and her family,” while another in FGD2 shared, “I tell her to strengthen her faith in God.” (FGD2, P6)

In some cases, participants mentioned referring mothers to psychologists or social workers for further support. However, these referral systems were described as poorly known, rarely utilized, and insufficiently integrated into routine care practices. Additionally, participants acknowledged significant gaps in formal training and the lack of resources, which hinder their ability to provide comprehensive and effective mental health care to mothers experiencing PPD.

#### 3.3.5. Importance of screening for PPD.

There was unanimous agreement across all FGDs on the importance of screening for PPD. Participants emphasized that early detection and intervention could significantly benefit mothers. In FGD2, a participant explained,

*“It’s important to screen to help the patients and guide them.” (FGD2, P5)*

Another participant in FGD5 added,

*“Screening is important because it will make things easier later.” (FGD5, P11)*

Many participants also highlighted the need for education and awareness as part of the screening process. In FGD3, one participant stated,

*“It’s very important. I think there should be more awareness about this topic. Women need to be educated about what to expect and how to prevent depression.” (FGD3, P8)*

Similarly, in FGD4, a participant suggested,

*“I think it’s important to start during pregnancy, preparing women for what to expect during delivery and after the baby is born. This will reduce their stress a lot.” (FGD4, C2)*

#### 3.3.6. Barriers to PPD screening.

Participants identified multiple barriers to the implementation of formal PPD screening within the hospital setting. Time constraints and heavy workload were consistently reported as major challenges. Nurses and midwives expressed concern about their limited capacity to integrate screening into routine care due to staff shortages and competing clinical responsibilities. One participant explained,

*“This is not our job as nurses. With only two or three nurses on duty, we are running around to finish our work. We can’t chase after patients to fill out a questionnaire”* (FGD5, P12). Similarly, another noted, *“This is not feasible. It will require time that we don’t have. Sometimes I don’t even have time to eat”* (FGD2, P6).

Low literacy and limited comprehension among patients were also perceived as significant obstacles to effective screening. Participants expressed concerns that some women may not fully understand screening questionnaires or may provide inaccurate responses. As one participant stated, *“The population we receive doesn’t have a good educational level. They might put anything on the questionnaire, or they might not understand the questions”* (FGD4, C3). Another added, *“There are people who don’t know how to read”* (FGD3, P10).

Cultural stigma surrounding mental health emerged as a prominent barrier. Participants reported that psychological distress and help-seeking were often associated with shame, mental instability, or being “crazy,” which discouraged both disclosure and acceptance of screening outcomes. One participant remarked,

*“People think that if you see a psychologist, you are crazy”* (FGD2, P5), while another explained, *“If we say a woman needs psychotherapy, her family will say she’s mentally unstable”* (FGD4, C1). Similar concerns were echoed by others who noted that seeking psychological care was viewed as shameful within some families (FGD2, P5).

Family and social dynamics were also identified as factors complicating screening and follow-up. Participants described situations where women faced pressure or abuse from family members that interfered with care. One participant shared,

*“We had a woman whose mother-in-law told her, ‘If you breastfeed the baby, you can’t come back home’”* (FGD4, C1). Another described a case of domestic violence, stating,

*“A woman’s husband beat her, and her family demanded a report from the gynecologist and a forensic doctor”* (FGD1, P4).

Finally, resource limitations, including the lack of dedicated personnel, were frequently mentioned. Participants emphasized that successful implementation of PPD screening would require assigning responsibility to trained staff rather than adding tasks to already overstretched nurses. One participant stated,

*“We need the right person in the right position. Without someone dedicated to this, it won’t work”* (FGD5, P12). A manager similarly emphasized, *“This project needs to be treated as an independent project, not part of the obstetric nurses’ workload”* (FGD4, C3).

#### 3.3.7. Facilitators and recommendations for implementing PPD screening.

Participants identified several facilitators to support the implementation of PPD screening, with dedicated personnel emerging as a central requirement. Many emphasized that screening should not be added to nurses’ existing workload but should be managed by trained professionals. One participant stated, *“We need someone dedicated to this mission, not the nurses”* (FGD3, P10), while another added, *“We need a specific person for this task, like a psychologist, who can take their time with each woman and provide follow-up”* (FGD1, P1).

Training and capacity building were also viewed as potentially valuable to successful implementation. Participants highlighted the need for education on PPD, the use of screening tools, and appropriate communication strategies. As one participant explained, *“We need training on how to ask the questions to get the best results and how to guide the women afterward”* (FGD2, P5). Another noted, *“We need training on PPD and how to use the screening tool”* (FGD4, C2, C3).

Managers emphasized the necessity of a clear protocol and referral pathway.

“We need a protocol from A to Z.” (FGD4, C3)

Participants further stressed the importance of integrating screening into routine care to improve its potential implementation and acceptability within existing clinical workflows. Suggestions included administering the screening during routine nursing care or scheduled postpartum encounters. One participant stated, *“The nurse could give the questionnaire to the patient during other care, like dressing changes, to save time”* (FGD1, P4), while another suggested that *“The screening could be done during the postpartum visit to reduce stigma”* (FGD4, C3).

Awareness raising among mothers and the community was perceived as critical to reducing stigma and improving acceptance of mental health services. A participant emphasized, *“We need awareness sessions for women about mental health and the importance of psychotherapy”* (FGD2, P5), while another suggested broader outreach through *“media, social networks, and advertisements”* (FGD2, P6).

Finally, participants highlighted the need for clear referral and follow-up mechanisms to ensure continuity of care for women with positive screening results. One participant suggested, *“The psychologist could come to the ward, educate the mothers, and do the screening herself”* (FGD3, P9), while another proposed coordination with primary care services, stating, *“Once a woman is screened, we notify the psychologists in the primary care center to evaluate her and schedule follow-ups”* (FGD2, P5).

## 4. Discussion

This study explored obstetric nurses’ knowledge, attitudes, and practices regarding PPD and identified key factors that may influence the potential implementation of systematic screening in a tertiary public hospital. Overall, participants expressed generally positive attitudes toward PPD screening and recognized the importance of maternal mental health. However, this positive attitude was not consistently matched by the knowledge, training, and institutional support required for effective implementation.

Although awareness of PPD was generally high, familiarity with validated screening tools and comprehensive knowledge of risk factors were limited. This finding suggests that PPD may be recognized conceptually without being fully integrated into evidence-based clinical practice. Similar gaps have been reported in other middle-income settings, where perinatal mental health receives limited attention in nursing education and continuing professional development programs [29,30]. The stronger recognition of symptoms compared with risk factors further indicates a tendency to focus on visible manifestations of distress rather than on underlying psychosocial vulnerabilities, potentially reducing opportunities for early identification and prevention [31].

Perceptions of PPD prevalence varied widely, suggesting that in the absence of systematic screening, case identification may rely largely on informal clinical assessments. Frontline staff tended to perceive PPD as nearly ubiquitous, likely reflecting cumulative exposure to social adversity, NICU admissions, and visible distress. In contrast, managerial estimates were more conservative and aligned with global prevalence figures [31], highlighting how role-based perspectives shape understanding of mental health burden. This divergence highlights the potential value of standardized screening in complementing informal clinical assessments with more systematic data collection [32,33].

Participants expressed generally positive attitudes toward routine PPD screening and acknowledged the important role of nurses in early detection. However, perceived barriers such as limited time, competing clinical priorities, and insufficient organizational support may hinder translation of these attitudes into practice. Consistent with previous research, favorable attitudes alone are insufficient to sustain screening programs without adequate training, clear protocols, and integration into routine workflows [29,34].

The findings also highlight important system-level challenges. Limited mental health training, uncertainty regarding referral pathways, staff shortages, short postpartum hospital stays, and persistent stigma surrounding mental health were identified as factors that may affect the implementation of systematic screening. Importantly, participants did not express resistance to screening itself; rather, they emphasized the need for structured processes, dedicated resources, and organizational support. This suggests that implementation efforts should focus on strengthening capacity and infrastructure rather than solely increasing awareness.

At the same time, the common agreement among participants regarding the value of screening and early intervention represents a potential facilitator for future implementation. The shared recognition among nurses and managers of the need for education, screening, and referral mechanisms indicates a potential receptiveness to introducing standardized approaches introducing standardized approaches to PPD care. Integrating validated screening tools into routine postnatal services, providing targeted training, and establishing clear referral pathways may help bridge the gap between awareness and practice.

### 4.1. Strengths and limitations

This study has several important strengths. First, the use of a mixed-methods design combining a KAP survey with focus group discussions allowed for a comprehensive exploration of PPD screening. Quantitative data provided measurable insights into nurses’ preparedness and practices, while qualitative findings offered deeper understanding of organizational, cultural, and contextual factors influencing implementation. Second, the study is original in its focus on the potential implementation of PPD screening within a Lebanese public hospital, a setting that remains underrepresented in the maternal mental health literature. The inclusion of both frontline obstetric nurses and managerial staff provided a more holistic perspective, capturing not only clinical realities but also organizational considerations relevant to implementation and sustainability.

Despite these strengths, several limitations should be acknowledged. The study was conducted in a single institution with a small census sample (n = 14), which limits the generalizability of the findings to other hospitals or healthcare settings. The reliance on self-administered questionnaires may also have introduced social desirability bias, potentially inflating positive attitudes or reported practices. Additionally, the study did not assess the acceptability of PPD screening from the mothers’ perspectives, which is a critical component of successful implementation. This study therefore opens avenues for future research and action, including expanding the evaluation to other Lebanese public and private hospitals to enable contextual comparisons, exploring maternal acceptance of and adherence to PPD screening, and assessing the long-term impact of systematic screening on maternal and infant well-being. It also underscores the importance of linking clinical practice with public health policy to ensure that PPD screening and management become integral components of maternal healthcare in Lebanon.

## 5. Conclusion

This study suggests a gap between the recognized importance of PPD screening, and the knowledge, preparedness, and institutional support required for its effective implementation. While obstetric nurses showed generally positive attitudes toward PPD screening and acknowledged their role in early detection, limitations in training, awareness of validated screening tools, and established referral processes may hinder consistent practice.

The findings suggest that integration of PPD screening into routine postnatal care requires targeted capacity-building, standardized screening protocols, clear referral pathways, and stronger organizational support. Building on the generally positive attitudes reported by participants may support future implementation efforts. However, as this study was conducted in a single tertiary public hospital with a small sample of obstetric nurses, the findings may not be generalizable to other healthcare settings in Lebanon. Further multicenter research involving larger and more diverse samples is needed to better understand implementation challenges and inform national strategies for PPD screening and management.

## Supporting information

S1 File KAP Questionnaire. (DOCX)

S2 File Interview topic guide. Feasibility of introducing a PPD screening tool.
(DOCX)

S3 File Data. (XLSX)

S1 Table Thematic analysis of focus group discussions. (DOCX)

## Abreviations:

PPD: Postpartum Depression
ACOG: American College of Obstetricians and Gynecologists
TGH: Tripoli Governmental Hospital
WHO: World Health Organization
EPDS: Edinburgh Postnatal Depression Scale
ICRC: International Committee of the Red Cross
NGO: Non-Governmental Organization
MSF: Médecins Sans Frontières (Doctors Without Borders)
KAP: Knowledge, Attitude, and Practice
