# Supplementary material for: Exploring perceptions, readiness, barriers, and facilitators related to the potential implementation of postpartum depression screening: A mixed-methods study in a Lebanese maternity setting
Source: PLoS One. 2026 Jul 30;21(7):e0354470. doi: 10.1371/journal.pone.0354470 (PMC13423026; doi:10.1371/journal.pone.0354470)
Supplement: S1 File — (DOCX) [file pone.0354470.s001.docx]

**S1 File: KAP Questionnaire**

**Dear Participant,**

Thank you for taking the time to complete this questionnaire. The aim of this study is to better understand the knowledge, attitudes, and practices of obstetrical nurses regarding postpartum depression and it's screening. Your participation is very important and will help improve the quality of maternal mental health care at Tripoli Governmental Hospital.

The questionnaire is anonymous, and your responses will remain confidential. Completing the questionnaire will take approximately 10–15 minutes.

Thank you again for your valuable contribution!

**Demographic information**

Years of clinical experience*

Less than 1 year

1-5 years

6-10 years

More than 10 years

**Knowledge**

1- Are you aware about the entity called postpartum depression?*

Yes

No

2- Have you heard about the term "baby blues"*

Yes

No

3- Is postpartum depression different from baby blues?*

Yes

No

4- Which of the following best describes the typical duration of the baby blues compared to postpartum depression?*

Baby blues last more than 6 weeks; PPD resolves within 3–5 days

Baby blues usually last up to 2 weeks; PPD persists beyond 2 weeks and may last for several  months

Both baby blues and PPD typically last the same duration—about 3 weeks

Baby blues last several months; PPD is limited to the first week postpartum

Other, specify

5-What are the risk factors of postpartum depression? (Select all that applies)*

Unplanned or unwanted pregnancy

Having a supportive partner

Financial insecurity

Preterm birth or infant health issues

Receiving epidural during labor

Other, specify

6- In your opinion, what are the symptoms of postpartum depression? (Select all that applies)*

Sleep disturbance

Persistent sadness, or low mood

Improved concentration and enhanced decision-making

Increased interest in social activities

Difficulty bonding with the baby

Other, specify

7- In your opinion what could be a management of postpartum depression? (Select all that applies)*

Stop breastfeeding

Psychotherapy

Antidepressants

No treatment needed symptoms will resolve by themselves

Other, specify

8- In your opinion, what can be the effect of untreated depression in perinatal period? (Select all that applies)*

Chronic depression and suicidal ideation

Poor emotional development in the baby

Lower risk of anxiety in the child

Improved maternal sleep due to reduced activity levels

Other, specify

9- In your opinion who is the best person to treat perinatal depression?*

Nurse/midwife

Obstetrician

Psychologist/psychiatrist

Social worker

No treatment required

Other, specify

10- Have you heard about any screening questionnaire for peri-natal depression?*

Yes

No

If Yes, please specify……..

**Attitude**

11- Postpartum depression is a serious condition that should be routinely screened in all postpartum women*

1- Strongly disagree

2- Disagree

3- Agree

4- Strongly agree

12- I feel confident identifying signs of postpartum depression in mothers*

1- Strongly disagree

2- Disagree

3- Agree

4- Strongly agree

13- Screening for postpartum depression should be part of routine postnatal care in the hospital*

1- Strongly disagree

2- Disagree

3- Agree

4- Strongly agree

14- Time constraints prevent healthcare providers from properly screening mothers for postpartum depression*

1- Strongly disagree

2- Disagree

3- Agree

4- Strongly agree

15- Nurses and midwives can play a key role in detecting postpartum depression early*

1- Strongly disagree

2- Disagree

3- Agree

4- Strongly agree

**Practice**

16- I ask postpartum mothers about their emotional wellbeing before discharge*

Always

Sometimes

Rarely

Never

17- I provide mothers with information about signs of postpartum depression before they leave the hospital*

Always

Sometimes

Rarely

Never

18- If I suspect a mother has postpartum depression, I discuss it with a doctor or supervisor*

Always

Sometimes

Rarely

Never

19- I have received training about mental health or postpartum depression during my professional career*

Yes

No

20- I feel I have enough knowledge and tools to support a mother showing signs of emotional distress*

Yes

No
