# Supplementary material for: Exploring perceptions, readiness, barriers, and facilitators related to the potential implementation of postpartum depression screening: A mixed-methods study in a Lebanese maternity setting
Source: PLoS One. 2026 Jul 30;21(7):e0354470. doi: 10.1371/journal.pone.0354470 (PMC13423026; doi:10.1371/journal.pone.0354470)
Supplement: S2 File — Feasibility of introducing a PPD screening tool. (DOCX) [file pone.0354470.s002.docx]

**Appendix 2: Interview Topic Guide: Feasibility of Introducing a PPD Screening Tool**

This guide will be used to facilitate focus group discussions with obstetrical nurses and key management staff to assess the feasibility of implementing a postpartum depression (PPD) screening tool at the Maternity Department. Questions will be open-ended and adapted based on findings from the quantitative phase (KAP survey). The main domains include:

**1. Introduction**

- Brief explanation of the session's purpose: to explore the feasibility of implementing a screening tool for postpartum depression (e.g., EPDS) in the postpartum ward.
- Explain that the discussion will inform decisions about practical implementation, training needs, and possible system changes.
- Emphasize that there are no right or wrong answers—just experiences and honest opinions.
- Ask for consent to record and reassure confidentiality.

**2. Awareness and Relevance of PPD Screening**

Explain postpartum depression risk factors: financial constraints, unwanted pregnancies, intimate partner violence, NICU admission, preterm labor…

- Do you think these risk factors are common in your context?
- In your opinion, how common is postpartum depression in the patients you see?
- What do you think about the importance of screening mothers for postpartum depression?

**3. Understanding Current Practices (Brief Section)**

- How do you currently identify or manage mothers who may be experiencing emotional distress?
- Do you feel confident recognizing signs of postpartum depression without a tool? Why or why not ?

**4. Feasibility of Screening Implementation**

- What do you think about introducing a formal screening tool for postpartum depression?

Start explaining about the presence of **EPDS (Edinburgh Postnatal Depression Scale)**: when, to whom, how much time, scoring.

- How feasible would it be to use a tool like the **EPDS (Edinburgh Postnatal Depression Scale)** in your daily work before patient’s discharge?
  - Prompt if needed: workload, time constraints, staff availability, documentation processes
- What support or resources would you need to implement this screening?

**5. Acceptability and Staff Buy-In**

- How do you think the rest of your team would react to adding PPD screening into routine care?
- What concerns might nurses have about implementing this?
- What would help improve staff acceptance and participation?

**6. Patient Considerations**

- How do you think mothers would respond to being screened for depression before discharge?
- Are there cultural or social factors that might make this more difficult or sensitive?

**7. Training and Capacity**

- What kind of training would you need to use and interpret a screening tool like EPDS?
- Have you received any prior training on maternal mental health or PPD?

**8. Barriers and Facilitators**

- What do you think are the main barriers to implementing a screening tool for PPD in your ward?
- What could help make it easier or more effective to introduce and use such a tool?

**9. System and Referral Considerations**

- If a mother screens positive, what steps do you think should follow?
- Are there existing referral systems or mental health support services that you are aware of?
- What improvements would be needed in the system to manage mothers who screen positive?

**10. Closing Questions**

- If you could suggest one thing to ensure successful screening for PPD in your ward, what would it be?
- Is there anything else you’d like to share on this topic?
