# Supplementary material for: Exploring perceptions, readiness, barriers, and facilitators related to the potential implementation of postpartum depression screening: A mixed-methods study in a Lebanese maternity setting
Source: PLoS One. 2026 Jul 30;21(7):e0354470. doi: 10.1371/journal.pone.0354470 (PMC13423026; doi:10.1371/journal.pone.0354470)
Supplement: S1 Table — (DOCX) [file pone.0354470.s004.docx]

**S1 Table: Thematic analysis of Focus Group Discussions**

| **Theme** | **Sub-theme** | **Initial Codes** | **Illustrative Quotes (Verbatims)** | **Group** |
| --- | --- | --- | --- | --- |
| **Theme 1: Perception of the prevalence and risk factors of PPD** | Prevalence | Very high estimation | “Even 100% of our patients.” (P6, FG2) | Nurses |
|  |  | Lower estimation | “I think it is around 20%.” (C1, FG4) | Managers |
|  | Risk factors | Violence, family constraints, baby’s sex, poverty | “Her husband beat her…” (P4, FG1) | Nurses |
| **Theme 2: Importance of screening** | Importance | Strong consensus | “It is certainly important.” (P11, FG5) | All |
|  | Prevention | Screening starting during pregnancy | “It is important to start during pregnancy.” (C2, FG4) | Managers |
| **Theme 3: Current identification of cases** | Observation | Behavioral signs (sadness, withdrawal, anxiety) | “We can tell from her behavior…” (P6, FG2) | Nurses |
|  | Family relationships | Controlling entourage | “Someone answers instead of her.” (C2, FG4) | Managers |
|  | Limitations | Risk of invisibility | “Some cases go unnoticed.” (C1, FG4) | Managers |
| **Theme 4: Current support practices** | Emotional support | Listening, advice, reassurance | “I talk to her and her family.” (P1, FG1) | Nurses |
|  | Religious approach | Turning to faith | “I tell her to strengthen her faith in God.” (P6, FG2) | Nurses |
|  | Referral | Limited resources | “The midwife refers her… to the primary care center.” (C1, FG4) | Managers |
| **Theme 5: Perceived feasibility of a formal tool (EPDS)** | Constraints | Lack of time, heavy workload | “I don’t even have time to eat.” (P6, FG2) | Nurses |
|  | Reliability | Completion by family members, illiteracy | “It won’t be the patient who fills it out.” (C1, FG4) | Managers |
|  | Neglect | Low adherence to brochures | “They leave them in the rooms.” (P8, FG3) | Nurses |
| **Theme 6: Barriers to implementation** | Organization | Work overload, insufficient staffing | “The medical record is less important than care.” (P1, FG1) | Nurses |
|  | Culture & stigma | Shame, taboos, fear of husband | “People say she is crazy.” (P5, FG2) | Nurses |
|  | Education level | Limited understanding | “They won’t understand the questions.” (C2, FG4) | Managers |
| **Theme 7: Support and resource needs** | Dedicated person | Psychologist, social worker | “This is not our job.” (P12, FG5) | Nurses |
|  | Clear system | Protocol, referral pathway | “We need a protocol… from A to Z.” (C3, FG4) | Managers |
|  | Coordination | Unawareness of MSF resources | “We didn’t know there were two MSF psychologists.” (P5, FG2) | Nurses |
| **Theme 8: Training needs** | PPD training | Insufficient knowledge | “We need sufficient information.” (P1, FG1) | Nurses |
|  | Communication | Knowing how to ask questions | “Training to ask the questions.” (P5, FG2) | Nurses |
|  | EPDS use | Score interpretation | “Someone must explain each question.” (C3, FG4) | Managers |
| **Theme 9: Acceptability and reactions of patients and staff** | Patients | Mixed reactions | “They will fill it out randomly.” (P12, FG5) | Nurses |
|  | Staff | Potential resistance | “They will say we barely have time.” (C1, FG4) | Managers |
