# Supplementary material for: Exploring perceptions, readiness, barriers, and facilitators related to the potential implementation of postpartum depression screening: A mixed-methods study in a Lebanese maternity setting
Source: PLoS One. 2026 Jul 30;21(7):e0354470. doi: 10.1371/journal.pone.0354470 (PMC13423026; doi:10.1371/journal.pone.0354470)
Supplement: Attachment — Submitted filename: Response to Reviewer.docx [file pone.0354470.s006.docx]

Dear Dr. Kattimani,

We are pleased to resubmit the revised version of Manuscript entitled: “***Exploring Perceptions, Readiness, Barriers, and Facilitators related to the potential implementation of Postpartum Depression Screening: A Mixed-Methods Study in a Lebanese Maternity Setting***”

Ref: Submission ID: PONE-D-26-07800

We would like to thank the editor and the reviewers for their constructive and insightful comments. We have carefully addressed all comments. All responses to the reviewers’ comments are provided in italics and prefaced by “Author response.” Modifications in the manuscript are track-changed. Please find below a summary of how we addressed the comments.

We sincerely appreciate the opportunity to resubmit and are enthusiastic about the possibility of publication.

Lama Al Hajj Sleiman, MSc

**Reviewer #1:**

1. Inconsistency regarding statistical analyses: The abstract states that “inferential analyses” were performed; however, only descriptive statistics (frequencies and percentages) are presented in the methods and results sections. If inferential analyses were not conducted, the abstract and methods sections should be revised accordingly. If such analyses were performed, they should be clearly described.

***Author’s Reply: Corrected***

2. Sample size and strength of interpretations: The study is based on a very small single-center sample (n=14). Nevertheless, some statements in the discussion and conclusion sections appear stronger than warranted by the available data. Expressions such as “institutional readiness,” “strong willingness,” and “persistent mismatch” should be framed more cautiously.

***Author’s Reply: We thank the reviewer for this important observation. In response, we have carefully revised the Discussion and Conclusion sections to temper overly strong wording and ensure that interpretations are consistent with the exploratory nature of the study. We have also revised the tone of interpretation to emphasize the exploratory and context-specific nature of the findings and to avoid overgeneralization.***

3. Validation of the KAP questionnaire: The validation process of the questionnaire should be described in greater detail. The authors should clarify which sections were adapted from previous instruments, whether pilot testing was conducted, and whether reliability measures (e.g., Cronbach’s alpha) or construct validity assessments were performed.

***Author’s Reply: We thank the reviewer for this important comment. The questionnaire was adapted from previously published studies assessing nurses’ and trainees’ knowledge, attitudes, and practices regarding postpartum depression screening and management (Kang et al., 2019; Hashim et al., 2022). Items were selected and modified to ensure relevance to the Lebanese maternity care context and alignment with the study objectives.***

***Face validity was assessed through independent review by two experienced midwives, who evaluated clarity, relevance, and comprehensibility. Minor revisions were incorporated based on their feedback. No formal pilot testing or psychometric validation (e.g., construct validity or internal consistency reliability testing) was conducted, as the instrument was used for descriptive purposes within a small total population sample.***

***Given the exploratory nature of the study and the limited sample size, the findings are intended to explore knowledge, attitudes, and practices rather than to validate the measurement properties of the instrument.***

4. Qualitative methodological rigor: Although Braun and Clarke’s framework is referenced, important qualitative rigor components such as thematic saturation, reflexivity, and researcher positionality are insufficiently described. Strengthening reporting in line with COREQ recommendations would improve the methodological quality of the manuscript.

***Author’s Reply:*** ***We reviewed the manuscript against the COREQ reporting guidelines and incorporated additional methodological details where applicable to improve the completeness and transparency of reporting. These revisions have been incorporated into the qualitative methods section, and the study limitations have been updated to acknowledge any remaining methodological constraints.***

5. Data availability statement: The current data availability statement may not fully comply with PLOS ONE data-sharing policies. The authors should clarify whether de-identified data may be accessible upon reasonable request or through controlled access procedures.

***Author’s Reply: De-identified data have been uploaded as Supporting Information. Qualitative excerpts supporting the findings are presented within the manuscript to illustrate and substantiate the identified themes.***

Framing of “feasibility”: The manuscript is framed as an evaluation of EPDS implementation feasibility; however, no actual pilot implementation, workflow testing, or implementation outcome assessment was performed. Therefore, the manuscript may be more accurately framed as an exploration of perceptions, readiness, barriers, and facilitators related to potential implementation.

***Author’s Reply: We thank the reviewer for this important observation. We agree that the term “feasibility” may imply that a pilot implementation, workflow testing, or formal assessment of implementation outcomes was conducted. As our study focused on exploring nurses’ knowledge, attitudes, and practices regarding PPD screening, as well as exploring perceived barriers, facilitators, and organizational readiness, we have revised the manuscript to more accurately reflect its scope.***

***Specifically, references to the “feasibility of EPDS implementation” have been replaced throughout the manuscript with wording that emphasizes the exploration of perceptions, readiness, barriers, and facilitators related to the potential implementation of the PPD Screening. Corresponding revisions were made to the title, abstract, objectives, methods, results, discussion, and conclusion sections to ensure consistency and to avoid implying that implementation feasibility was directly evaluated.***

Minor Revisions: The phrase “low- and middle-income countries” should be corrected to “middle-income countries.”

***Author’s Reply: Corrected***

The categories “Good practice” and “Poor practice” may sound overly normative and could be replaced with more neutral terminology such as “consistent/inconsistent practice.”
***Author’s Reply: We thank the reviewer for this helpful suggestion. We have revised the terminology throughout the manuscript and replaced these categories with the more neutral descriptors “Consistent practice” and “Inconsistent practice.” Corresponding changes have been made in the Methods, Results, tables, and relevant text to ensure consistency.***

Some parts of the discussion section repeat findings already presented in the results and could be condensed.

***Author’s Reply: We have revised the Discussion section to reduce repetition of findings already reported in the Results section.***

The phrase “subjective judgment” may be replaced with a more neutral expression such as “informal clinical assessment.”

***Author’s Reply: Corrected***

The single-center nature of the study and its limited generalizability should be emphasized more clearly in the conclusion section.

***Author’s Reply: We have revised the conclusion to explicitly acknowledge these limitations and to emphasize that the results should be interpreted with caution. We also added a recommendation for future multicenter studies with larger and more diverse samples to validate the findings and support broader implementation of PPD screening strategies.***

Overall Evaluation: The manuscript addresses an important public health issue and has publishable scientific potential. However, revisions are necessary to improve methodological transparency, compliance with data-sharing policies, and the framing of interpretations and implementation claims.

Reviewer #2: General Comment: You can add co-authors from your team.

***Author’s Reply: Thank you for the suggestion. However, this study was conducted by only two researchers: one epidemiologist and one experienced midwife, who were fully involved in all stages of the work, including study design, data collection, analysis, and manuscript writing. Therefore, no additional co-authors are included.***

Abstract: Please add statistics in the Results.

***Author’s Reply: Added***

Method: Give the justification of Study design and why was only one setting chosen?

***Author’s Reply: We thank the reviewer for this important comment. Justifications were added to the methods section***

Quantitative Component is very weak in methodology.

***Author’s Reply: We thank the reviewer for this observation. The quantitative component in the has been revised. We clarified the cross-sectional design, justified the use of total population sampling (n=14), and provided a more detailed description of the questionnaire development process, including adaptation from previously published instruments, translation procedures, face validity assessment, and the absence of formal psychometric testing given the exploratory nature of the study. We also clarified that the quantitative analysis was strictly descriptive, using frequencies and percentages, and ensured consistency between the Methods and Results sections.***

Sample Size is not described properly. n=14 is not justifiable.

***Author’s Reply: The study included all obstetric nurses working in the maternity setting at TGH during the study period; therefore, the sample (n=14) represents a total population sampling (census) rather than a selected or partial sample.***

***We have revised the Methods section to clearly state that all eligible nurses in the institution were invited and included, and that no sampling was done due to the small and fixed workforce size. We have also clarified this in the manuscript to improve transparency regarding the study design and sampling approach.***

Operational definitions are missing.

***Author’s Reply: Thank you for this valuable suggestion. The definition of PPD is provided in the Introduction section. In the Methods section, we focused on defining and describing the assessment of the knowledge, attitudes, and practices variables, as these constitute the main study outcomes.***

Inferential statistics were mentioned but not reported in the results.

***Author’s Reply: We thank the reviewer for this important observation. This was an oversight in the reporting of the quantitative analysis. In the revised manuscript, we have corrected this inconsistency by removing references to inferential statistics and ensuring that the Methods section accurately reflects the descriptive nature of the quantitative analysis performed. The Results section has been aligned accordingly to only descriptive statistics.***
