# Supplementary material for: Exploring perceptions, readiness, barriers, and facilitators related to the potential implementation of postpartum depression screening: A mixed-methods study in a Lebanese maternity setting
Source: PLoS One. 2026 Jul 30;21(7):e0354470. doi: 10.1371/journal.pone.0354470 (PMC13423026; doi:10.1371/journal.pone.0354470)
Supplement: Peer review history

# Peer review: Decision Letter 0

**DOI:** 10.1371/journal.pone.0354470.r001

**Authors:** Shivanand Kattimani

27 May 2026

PONE-D-26-07800

Exploring the Feasibility of Postpartum Depression Screening: A Mixed-Methods Study in a Lebanese Maternity Setting

PLOS One

Dear Dr. El Haj Sleiman,

Thank you for submitting your manuscript to PLOS ONE. After careful consideration, we feel that it has merit but does not fully meet PLOS ONE’s publication criteria as it currently stands. Therefore, we invite you to submit a revised version of the manuscript that addresses the points raised during the review process.

Please submit your revised manuscript by Jul 11 2026 11:59PM. If you will need more time than this to complete your revisions, please reply to this message or contact the journal office at plosone@plos.org. When you're ready to submit your revision, log on to https://www.editorialmanager.com/pone/ and select the 'Submissions Needing Revision' folder to locate your manuscript file.

Please include the following items when submitting your revised manuscript:

- A letter that responds to each point raised by the academic editor and reviewer(s). You should upload this letter as a separate file labeled 'Response to Reviewers'.
- A marked-up copy of your manuscript that highlights changes made to the original version. You should upload this as a separate file labeled 'Revised Manuscript with Track Changes'.
- An unmarked version of your revised paper without tracked changes. You should upload this as a separate file labeled 'Manuscript'.

If you would like to make changes to your financial disclosure, please include your updated statement in your cover letter. Guidelines for resubmitting your figure files are available below the reviewer comments at the end of this letter.

If applicable, we recommend that you deposit your laboratory protocols in protocols.io to enhance the reproducibility of your results. Protocols.io assigns your protocol its own identifier (DOI) so that it can be cited independently in the future. For instructions see: https://journals.plos.org/plosone/s/submission-guidelines#loc-laboratory-protocols. Additionally, PLOS ONE offers an option for publishing peer-reviewed Lab Protocol articles, which describe protocols hosted on protocols.io. Read more information on sharing protocols at https://plos.org/protocols?utm_medium=editorial-email&utm_source=authorletters&utm_campaign=protocols.

As the corresponding author, your ORCID iD is verified in the submission system and will appear in the published article. PLOS supports the use of ORCID, and we encourage all coauthors to register for an ORCID iD and use it as well. Please encourage your coauthors to verify their ORCID iD within the submission system before final acceptance, as unverified ORCID iDs will not appear in the published article. *Only*  the individual author can complete the verification step; PLOS staff *cannot*  verify ORCID iDs on behalf of authors.

We look forward to receiving your revised manuscript.

Kind regards,

Shivanand Kattimani

Academic Editor

PLOS One

**Journal Requirements:** When submitting your revision, we need you to address these additional requirements.

1. Please ensure that your manuscript meets PLOS ONE's style requirements, including those for file naming. The PLOS ONE style templates can be found at

https://journals.plos.org/plosone/s/file?id=wjVg/PLOSOne_formatting_sample_main_body.pdf and

https://journals.plos.org/plosone/s/file?id=ba62/PLOSOne_formatting_sample_title_authors_affiliations.pdf

2. We note that you have indicated that there are restrictions to data sharing for this study. PLOS only allows data to be available upon request if there are legal or ethical restrictions on sharing data publicly. For more information on unacceptable data access restrictions, please see http://journals.plos.org/plosone/s/data-availability#loc-unacceptable-data-access-restrictions.

Before we proceed with your manuscript, please address the following prompts:

a) If there are ethical or legal restrictions on sharing a de-identified data set, please explain them in detail (e.g., data contain potentially identifying or sensitive patient information, data are owned by a third-party organization, etc.) and who has imposed them (e.g., a Research Ethics Committee or Institutional Review Board, etc.). Please also provide contact information for a data access committee, ethics committee, or other institutional body to which data requests may be sent.

b) If there are no restrictions, please upload the minimal anonymized data set necessary to replicate your study findings to a stable, public repository and provide us with the relevant URLs, DOIs, or accession numbers. For a list of recommended repositories, please see

https://journals.plos.org/plosone/s/recommended-repositories. You also have the option of uploading the data as Supporting Information files, but we would recommend depositing data directly to a data repository if possible.

We will update your Data Availability statement on your behalf to reflect the information you provide.

3. Please include your full ethics statement in the ‘Methods’ section of your manuscript file. In your statement, please include the full name of the IRB or ethics committee who approved or waived your study, as well as whether or not you obtained informed written or verbal consent. If consent was waived for your study, please include this information in your statement as well.

4. Please upload a new copy of Figure 1 as the detail is not clear. Please follow the link for more information:  https://journals.plos.org/plosone/s/figures

5. Please include captions for your Supporting Information files at the end of your manuscript, and update any in-text citations to match accordingly. Please see our Supporting Information guidelines for more information: http://journals.plos.org/plosone/s/supporting-information.

6. If the reviewer comments include a recommendation to cite specific previously published works, please review and evaluate these publications to determine whether they are relevant and should be cited. There is no requirement to cite these works unless the editor has indicated otherwise.

7. Please review your reference list to ensure that it is complete and correct. If you have cited papers that have been retracted, please include the rationale for doing so in the manuscript text, or remove these references and replace them with relevant current references. Any changes to the reference list should be mentioned in the rebuttal letter that accompanies your revised manuscript. If you need to cite a retracted article, indicate the article’s retracted status in the References list and also include a citation and full reference for the retraction notice.

Reviewers' comments:

Reviewer's Responses to Questions

**Comments to the Author**

1. Is the manuscript technically sound, and do the data support the conclusions?

The manuscript must describe a technically sound piece of scientific research with data that supports the conclusions. Experiments must have been conducted rigorously, with appropriate controls, replication, and sample sizes. The conclusions must be drawn appropriately based on the data presented.

Reviewer #1: Yes

Reviewer #2: Yes

**********

2. Has the statistical analysis been performed appropriately and rigorously?

Reviewer #1: Yes

Reviewer #2: Yes

**********

3. Have the authors made all data underlying the findings in their manuscript fully available?

The PLOS Data policy requires authors to make all data underlying the findings described in their manuscript fully available without restriction, with rare exception (please refer to the Data Availability Statement in the manuscript PDF file). The data should be provided as part of the manuscript or its supporting information, or deposited to a public repository. For example, in addition to summary statistics, the data points behind means, medians and variance measures should be available. If there are restrictions on publicly sharing data—e.g. participant privacy or use of data from a third party—those must be specified.

Reviewer #1: No

Reviewer #2: Yes

**********

4. Is the manuscript presented in an intelligible fashion and written in standard English?

PLOS ONE does not copyedit accepted manuscripts, so the language in submitted articles must be clear, correct, and unambiguous. Any typographical or grammatical errors should be corrected at revision, so please note any specific errors here.

Reviewer #1: Yes

Reviewer #2: Yes

**********

5. Review Comments to the Author

Please use the space provided to explain your answers to the questions above. You may also include additional comments for the author, including concerns about dual publication, research ethics, or publication ethics. (Please upload your review as an attachment if it exceeds 20,000 characters)

Reviewer #1: This manuscript addresses an important and timely topic by evaluating the feasibility of postpartum depression (PPD) screening in a Lebanese public hospital setting. The mixed-methods design is appropriate for the research question, and the focus group discussions provide valuable contextual insights. However, several issues should be addressed to improve methodological transparency and strengthen the scientific rigor of the study.

Major Revisions

Inconsistency regarding statistical analyses

The abstract states that “inferential analyses” were performed; however, only descriptive statistics (frequencies and percentages) are presented in the methods and results sections. If inferential analyses were not conducted, the abstract and methods sections should be revised accordingly. If such analyses were performed, they should be clearly described.

Sample size and strength of interpretations

The study is based on a very small single-center sample (n=14). Nevertheless, some statements in the discussion and conclusion sections appear stronger than warranted by the available data. Expressions such as “institutional readiness,” “strong willingness,” and “persistent mismatch” should be framed more cautiously.

Validation of the KAP questionnaire

The validation process of the questionnaire should be described in greater detail. The authors should clarify which sections were adapted from previous instruments, whether pilot testing was conducted, and whether reliability measures (e.g., Cronbach’s alpha) or construct validity assessments were performed.

Qualitative methodological rigor

Although Braun and Clarke’s framework is referenced, important qualitative rigor components such as thematic saturation, reflexivity, and researcher positionality are insufficiently described. Strengthening reporting in line with COREQ recommendations would improve the methodological quality of the manuscript.

Data availability statement

The current data availability statement may not fully comply with PLOS ONE data-sharing policies. The authors should clarify whether de-identified data may be accessible upon reasonable request or through controlled access procedures.

Framing of “feasibility”

The manuscript is framed as an evaluation of EPDS implementation feasibility; however, no actual pilot implementation, workflow testing, or implementation outcome assessment was performed. Therefore, the manuscript may be more accurately framed as an exploration of perceptions, readiness, barriers, and facilitators related to potential implementation.

Minor Revisions

The phrase “low- and middleincome countries” should be corrected to “middle-income countries.”

The categories “Good practice” and “Poor practice” may sound overly normative and could be replaced with more neutral terminology such as “consistent/inconsistent practice.”

Some parts of the discussion section repeat findings already presented in the results and could be condensed.

The phrase “subjective judgment” may be replaced with a more neutral expression such as “informal clinical assessment.”

The single-center nature of the study and its limited generalizability should be emphasized more clearly in the conclusion section.

Overall Evaluation

The manuscript addresses an important public health issue and has publishable scientific potential. However, revisions are necessary to improve methodological transparency, compliance with data-sharing policies, and the framing of interpretations and implementation claims.

Reviewer #2: General Comment: You can add co-authors from your team.

Abstract: Please add statistics in the Results.

Method: Give the justification of Study design and why only one setting was chosen?

Quantitative Component is very weak in methodology.

Sample Size not described properly. n=14 is not justifiable.

Operational definitions are missing.

Inferential statistics was mentioned but not reported in the results.

**********

6. PLOS authors have the option to publish the peer review history of their article (what does this mean?). If published, this will include your full peer review and any attached files.

If you choose “no”, your identity will remain anonymous but your review may still be made public.

**Do you want your identity to be public for this peer review?**  For information about this choice, including consent withdrawal, please see our Privacy Policy.

Reviewer #1: No

Reviewer #2: No

**********

[NOTE: If reviewer comments were submitted as an attachment file, they will be attached to this email and accessible via the submission site. Please log into your account, locate the manuscript record, and check for the action link "View Attachments". If this link does not appear, there are no attachment files.]

To ensure your figures meet our technical requirements, please review our figure guidelines: https://journals.plos.org/plosone/s/figures

You may also use PLOS’s free figure tool, NAAS, to help you prepare publication quality figures: https://journals.plos.org/plosone/s/figures#loc-tools-for-figure-preparation.

NAAS will assess whether your figures meet our technical requirements by comparing each figure against our figure specifications.

# Author comment: Author response to Decision Letter 1

**DOI:** 10.1371/journal.pone.0354470.r002

8 Jun 2026

Dear Dr. Kattimani,

We are pleased to resubmit the revised version of Manuscript entitled: “Exploring Perceptions, Readiness, Barriers, and Facilitators related to the potential implementation of Postpartum Depression Screening: A Mixed-Methods Study in a Lebanese Maternity Setting”

Ref: Submission ID: PONE-D-26-07800

We would like to thank the editor and the reviewers for their constructive and insightful comments. We have carefully addressed all comments. All responses to the reviewers’ comments are provided in italics and prefaced by “Author response.” Modifications in the manuscript are track-changed. Please find below a summary of how we addressed the comments.

We sincerely appreciate the opportunity to resubmit and are enthusiastic about the possibility of publication.

Lama Al Hajj Sleiman, MSc

Reviewer #1:

1. Inconsistency regarding statistical analyses: The abstract states that “inferential analyses” were performed; however, only descriptive statistics (frequencies and percentages) are presented in the methods and results sections. If inferential analyses were not conducted, the abstract and methods sections should be revised accordingly. If such analyses were performed, they should be clearly described.

Author’s Reply: Corrected

2. Sample size and strength of interpretations: The study is based on a very small single-center sample (n=14). Nevertheless, some statements in the discussion and conclusion sections appear stronger than warranted by the available data. Expressions such as “institutional readiness,” “strong willingness,” and “persistent mismatch” should be framed more cautiously.

Author’s Reply: We thank the reviewer for this important observation. In response, we have carefully revised the Discussion and Conclusion sections to temper overly strong wording and ensure that interpretations are consistent with the exploratory nature of the study. We have also revised the tone of interpretation to emphasize the exploratory and context-specific nature of the findings and to avoid overgeneralization.

3. Validation of the KAP questionnaire: The validation process of the questionnaire should be described in greater detail. The authors should clarify which sections were adapted from previous instruments, whether pilot testing was conducted, and whether reliability measures (e.g., Cronbach’s alpha) or construct validity assessments were performed.

Author’s Reply: We thank the reviewer for this important comment. The questionnaire was adapted from previously published studies assessing nurses’ and trainees’ knowledge, attitudes, and practices regarding postpartum depression screening and management (Kang et al., 2019; Hashim et al., 2022). Items were selected and modified to ensure relevance to the Lebanese maternity care context and alignment with the study objectives.

Face validity was assessed through independent review by two experienced midwives, who evaluated clarity, relevance, and comprehensibility. Minor revisions were incorporated based on their feedback. No formal pilot testing or psychometric validation (e.g., construct validity or internal consistency reliability testing) was conducted, as the instrument was used for descriptive purposes within a small total population sample.

Given the exploratory nature of the study and the limited sample size, the findings are intended to explore knowledge, attitudes, and practices rather than to validate the measurement properties of the instrument.

4. Qualitative methodological rigor: Although Braun and Clarke’s framework is referenced, important qualitative rigor components such as thematic saturation, reflexivity, and researcher positionality are insufficiently described. Strengthening reporting in line with COREQ recommendations would improve the methodological quality of the manuscript.

Author’s Reply: We reviewed the manuscript against the COREQ reporting guidelines and incorporated additional methodological details where applicable to improve the completeness and transparency of reporting. These revisions have been incorporated into the qualitative methods section, and the study limitations have been updated to acknowledge any remaining methodological constraints.

5. Data availability statement: The current data availability statement may not fully comply with PLOS ONE data-sharing policies. The authors should clarify whether de-identified data may be accessible upon reasonable request or through controlled access procedures.

Author’s Reply: De-identified data have been uploaded as Supporting Information. Qualitative excerpts supporting the findings are presented within the manuscript to illustrate and substantiate the identified themes.

6- Framing of “feasibility”: The manuscript is framed as an evaluation of EPDS implementation feasibility; however, no actual pilot implementation, workflow testing, or implementation outcome assessment was performed. Therefore, the manuscript may be more accurately framed as an exploration of perceptions, readiness, barriers, and facilitators related to potential implementation.

Author’s Reply: We thank the reviewer for this important observation. We agree that the term “feasibility” may imply that a pilot implementation, workflow testing, or formal assessment of implementation outcomes was conducted. As our study focused on exploring nurses’ knowledge, attitudes, and practices regarding PPD screening, as well as exploring perceived barriers, facilitators, and organizational readiness, we have revised the manuscript to more accurately reflect its scope.

Specifically, references to the “feasibility of EPDS implementation” have been replaced throughout the manuscript with wording that emphasizes the exploration of perceptions, readiness, barriers, and facilitators related to the potential implementation of the PPD Screening. Corresponding revisions were made to the title, abstract, objectives, methods, results, discussion, and conclusion sections to ensure consistency and to avoid implying that implementation feasibility was directly evaluated.

7- Minor Revisions: The phrase “low- and middle-income countries” should be corrected to “middle-income countries.”

Author’s Reply: Corrected

8- The categories “Good practice” and “Poor practice” may sound overly normative and could be replaced with more neutral terminology such as “consistent/inconsistent practice.”

Author’s Reply: We thank the reviewer for this helpful suggestion. We have revised the terminology throughout the manuscript and replaced these categories with the more neutral descriptors “Consistent practice” and “Inconsistent practice.” Corresponding changes have been made in the Methods, Results, tables, and relevant text to ensure consistency.

9- Some parts of the discussion section repeat findings already presented in the results and could be condensed.

Author’s Reply: We have revised the Discussion section to reduce repetition of findings already reported in the Results section.

10- The phrase “subjective judgment” may be replaced with a more neutral expression such as “informal clinical assessment.”

Author’s Reply: Corrected

11- The single-center nature of the study and its limited generalizability should be emphasized more clearly in the conclusion section.

Author’s Reply: We have revised the conclusion to explicitly acknowledge these limitations and to emphasize that the results should be interpreted with caution. We also added a recommendation for future multicenter studies with larger and more diverse samples to validate the findings and support broader implementation of PPD screening strategies.

Reviewer #2:

1- General Comment: You can add co-authors from your team.

Author’s Reply: Thank you for the suggestion. However, this study was conducted by only two researchers: one epidemiologist and one experienced midwife, who were fully involved in all stages of the work, including study design, data collection, analysis, and manuscript writing. Therefore, no additional co-authors are included.

2- Abstract: Please add statistics in the Results.

Author’s Reply: Added

3- Method: Give the justification of Study design and why was only one setting chosen?

Author’s Reply: We thank the reviewer for this important comment. Justifications were added to the methods section

4- Quantitative Component is very weak in methodology.

Author’s Reply: We thank the reviewer for this observation. The quantitative component in the has been revised. We clarified the cross-sectional design, justified the use of total population sampling (n=14), and provided a more detailed description of the questionnaire development process, including adaptation from previously published instruments, translation procedures, face validity assessment, and the absence of formal psychometric testing given the exploratory nature of the study. We also clarified that the quantitative analysis was strictly descriptive, using frequencies and percentages, and ensured consistency between the Methods and Results sections.

5- Sample Size is not described properly. n=14 is not justifiable.

Author’s Reply: The study included all obstetric nurses working in the maternity setting at TGH during the study period; therefore, the sample (n=14) represents a total population sampling (census) rather than a selected or partial sample.

We have revised the Methods section to clearly state that all eligible nurses in the institution were invited and included, and that no sampling was done due to the small and fixed workforce size. We have also clarified this in the manuscript to improve transparency regarding the study design and sampling approach.

6- Operational definitions are missing.

Author’s Reply: Thank you for this valuable suggestion. The definition of PPD is provided in the Introduction section. In the Methods section, we focused on defining and describing the assessment of the knowledge, attitudes, and practices variables, as these constitute the main study outcomes.

7- Inferential statistics were mentioned but not reported in the results.

Author’s Reply: We thank the reviewer for this important observation. This was an oversight in the reporting of the quantitative analysis. In the revised manuscript, we have corrected this inconsistency by removing references to inferential statistics and ensuring that the Methods section accurately reflects the descriptive nature of the quantitative analysis performed. The Results section has been aligned accordingly to only descriptive statistics.

Attachment Submitted filename: Response to Reviewer.docx

# Peer review: Decision Letter 1

**DOI:** 10.1371/journal.pone.0354470.r003

**Authors:** Shivanand Kattimani

24 Jun 2026

PONE-D-26-07800R1

Exploring Perceptions, Readiness, Barriers, and Facilitators related to the potential implementation of Postpartum Depression Screening: A Mixed-Methods Study in a Lebanese Maternity Setting

PLOS One

Dear Dr. El Haj Sleiman,

Thank you for submitting your manuscript to PLOS ONE. After careful consideration, we feel that it has merit but does not fully meet PLOS ONE’s publication criteria as it currently stands. Therefore, we invite you to submit a revised version of the manuscript that addresses the points raised during the review process.

Please submit your revised manuscript by Aug 08 2026 11:59PM. If you will need more time than this to complete your revisions, please reply to this message or contact the journal office at plosone@plos.org. When you're ready to submit your revision, log on to https://www.editorialmanager.com/pone/ and select the 'Submissions Needing Revision' folder to locate your manuscript file.

Please include the following items when submitting your revised manuscript:

- A letter that responds to each point raised by the academic editor and reviewer(s). You should upload this letter as a separate file labeled 'Response to Reviewers'.
- A marked-up copy of your manuscript that highlights changes made to the original version. You should upload this as a separate file labeled 'Revised Manuscript with Track Changes'.
- An unmarked version of your revised paper without tracked changes. You should upload this as a separate file labeled 'Manuscript'.

If you would like to make changes to your financial disclosure, please include your updated statement in your cover letter. Guidelines for resubmitting your figure files are available below the reviewer comments at the end of this letter.

If applicable, we recommend that you deposit your laboratory protocols in protocols.io to enhance the reproducibility of your results. Protocols.io assigns your protocol its own identifier (DOI) so that it can be cited independently in the future. For instructions see: https://journals.plos.org/plosone/s/submission-guidelines#loc-laboratory-protocols. Additionally, PLOS ONE offers an option for publishing peer-reviewed Lab Protocol articles, which describe protocols hosted on protocols.io. Read more information on sharing protocols at https://plos.org/protocols?utm_medium=editorial-email&utm_source=authorletters&utm_campaign=protocols.

As the corresponding author, your ORCID iD is verified in the submission system and will appear in the published article. PLOS supports the use of ORCID, and we encourage all coauthors to register for an ORCID iD and use it as well. Please encourage your coauthors to verify their ORCID iD within the submission system before final acceptance, as unverified ORCID iDs will not appear in the published article. *Only*  the individual author can complete the verification step; PLOS staff *cannot*  verify ORCID iDs on behalf of authors.

We look forward to receiving your revised manuscript.

Kind regards,

Shivanand Kattimani

Academic Editor

PLOS One

Journal Requirements:

1. If the reviewer comments include a recommendation to cite specific previously published works, please review and evaluate these publications to determine whether they are relevant and should be cited. There is no requirement to cite these works unless the editor has indicated otherwise.

2. Please review your reference list to ensure that it is complete and correct. If you have cited papers that have been retracted, please include the rationale for doing so in the manuscript text, or remove these references and replace them with relevant current references. Any changes to the reference list should be mentioned in the rebuttal letter that accompanies your revised manuscript. If you need to cite a retracted article, indicate the article’s retracted status in the References list and also include a citation and full reference for the retraction notice.

Reviewers' comments:

Reviewer's Responses to Questions

**Comments to the Author**

1. If the authors have adequately addressed your comments raised in a previous round of review and you feel that this manuscript is now acceptable for publication, you may indicate that here to bypass the “Comments to the Author” section, enter your conflict of interest statement in the “Confidential to Editor” section, and submit your "Accept" recommendation.

Reviewer #1: All comments have been addressed

**********

2. Is the manuscript technically sound, and do the data support the conclusions?

The manuscript must describe a technically sound piece of scientific research with data that supports the conclusions. Experiments must have been conducted rigorously, with appropriate controls, replication, and sample sizes. The conclusions must be drawn appropriately based on the data presented.

Reviewer #1: Partly

**********

3. Has the statistical analysis been performed appropriately and rigorously?

Reviewer #1: Yes

**********

4. Have the authors made all data underlying the findings in their manuscript fully available?

The PLOS Data policy requires authors to make all data underlying the findings described in their manuscript fully available without restriction, with rare exception (please refer to the Data Availability Statement in the manuscript PDF file). The data should be provided as part of the manuscript or its supporting information, or deposited to a public repository. For example, in addition to summary statistics, the data points behind means, medians and variance measures should be available. If there are restrictions on publicly sharing data—e.g. participant privacy or use of data from a third party—those must be specified.

Reviewer #1: No

**********

5. Is the manuscript presented in an intelligible fashion and written in standard English?

PLOS ONE does not copyedit accepted manuscripts, so the language in submitted articles must be clear, correct, and unambiguous. Any typographical or grammatical errors should be corrected at revision, so please note any specific errors here.

Reviewer #1: Yes

**********

6. Review Comments to the Author

Please use the space provided to explain your answers to the questions above. You may also include additional comments for the author, including concerns about dual publication, research ethics, or publication ethics. (Please upload your review as an attachment if it exceeds 20,000 characters)

Reviewer #1: Thank you for your revised submission. The authors have adequately addressed several of the previously raised concerns, including the reframing of "feasibility" terminology, the addition of reflexivity and researcher positionality statements, the clarification of data availability, and the adoption of neutral practice categories. These revisions are appreciated.

However, the following issues remain unresolved and must be corrected before the manuscript can be accepted:

1. Overly strong interpretive language (Major Revision #2): Despite the authors' response indicating that the Discussion and Conclusion sections were revised to temper overly strong wording, several problematic expressions remain in the manuscript. Specifically, the phrases "institutional readiness" (Methods/Abstract), "persistent mismatch," and "strong attitudinal support" are still present in the Discussion section. Given the single-center, small sample size (n=14), these expressions are not warranted and must be replaced with more cautious, exploratory language throughout.

2. Inconsistent correction of "low- and middle-income countries" (Minor Revision #7): The requested change from "low- and middle-income countries" to "middle-income countries" has only been applied in select locations. The phrase "low- and middle-income" remains unchanged in multiple sections, including the Abstract, Introduction, and Discussion. Authors must ensure this correction is applied consistently throughout the entire manuscript.

3. "Subjective judgment" not replaced (Minor Revision #10): The phrase "subjective judgment" remains in the manuscript and has not been replaced with the suggested neutral expression "informal clinical assessment" as indicated in the authors' response.

The authors are asked to carefully revise the manuscript to address all three points above and to ensure that all previously committed changes are consistently applied throughout the full text.

**********

7. PLOS authors have the option to publish the peer review history of their article (what does this mean?). If published, this will include your full peer review and any attached files.

If you choose “no”, your identity will remain anonymous but your review may still be made public.

**Do you want your identity to be public for this peer review?**  For information about this choice, including consent withdrawal, please see our Privacy Policy.

Reviewer #1: No

**********

[NOTE: If reviewer comments were submitted as an attachment file, they will be attached to this email and accessible via the submission site. Please log into your account, locate the manuscript record, and check for the action link "View Attachments". If this link does not appear, there are no attachment files.]

To ensure your figures meet our technical requirements, please review our figure guidelines: https://journals.plos.org/plosone/s/figures

You may also use PLOS’s free figure tool, NAAS, to help you prepare publication quality figures: https://journals.plos.org/plosone/s/figures#loc-tools-for-figure-preparation.

NAAS will assess whether your figures meet our technical requirements by comparing each figure against our figure specifications.

# Author comment: Author response to Decision Letter 2

**DOI:** 10.1371/journal.pone.0354470.r004

25 Jun 2026

Reply to Reviewer

1. Overly strong interpretive language (Major Revision #2): Despite the authors' response indicating that the Discussion and Conclusion sections were revised to temper overly strong wording, several problematic expressions remain in the manuscript. Specifically, the phrases "institutional readiness" (Methods/Abstract), "persistent mismatch," and "strong attitudinal support" are still present in the Discussion section. Given the single-center, small sample size (n=14), these expressions are not warranted and must be replaced with more cautious, exploratory language throughout.

Author’s Reply: We thank the reviewer for this important observation. We have carefully reviewed the manuscript and replaced these terms with more cautious language throughout the Abstract, Methods, Results, Discussion, and Conclusion.

2. Inconsistent correction of "low- and middle-income countries" (Minor Revision #7): The requested change from "low- and middle-income countries" to "middle-income countries" has only been applied in select locations. The phrase "low- and middle-income" remains unchanged in multiple sections, including the Abstract, Introduction, and Discussion. Authors must ensure this correction is applied consistently throughout the entire manuscript.

Author’s Reply: We thank the reviewer for identifying this oversight. We have conducted a comprehensive review of the entire manuscript and corrected all remaining references to "low- and middle-income countries" to ensure consistency with the study context and reviewer recommendation. The terminology has now been revised throughout the Abstract, Introduction, Discussion, and any other relevant sections.

3. "Subjective judgment" not replaced (Minor Revision #10): The phrase "subjective judgment" remains in the manuscript and has not been replaced with the suggested neutral expression "informal clinical assessment" as indicated in the authors' response.

Author’s reply: We thank the reviewer for highlighting this omission. The relevant text has been revised to replace references to subjective judgment or subjective estimation with the term "informal clinical assessment."

# Peer review: Decision Letter 2

**DOI:** 10.1371/journal.pone.0354470.r005

**Authors:** Shivanand Kattimani

8 Jul 2026

Exploring Perceptions, Readiness, Barriers, and Facilitators related to the potential implementation of Postpartum Depression Screening: A Mixed-Methods Study in a Lebanese Maternity Setting

PONE-D-26-07800R2

Dear Dr. El Haj Sleiman,

We’re pleased to inform you that your manuscript has been judged scientifically suitable for publication and will be formally accepted for publication once it meets all outstanding technical requirements.

Within one week, you’ll receive an e-mail detailing the required amendments. When these have been addressed, you’ll receive a formal acceptance letter and your manuscript will be scheduled for publication.

An invoice will be generated when your article is formally accepted. Please note, if your institution has a publishing partnership with PLOS and your article meets the relevant criteria, all or part of your publication costs will be covered. Please make sure your user information is up-to-date by logging into Editorial Manager at Editorial Manager® and clicking the ‘Update My Information' link at the top of the page. For questions related to billing, please contact billing support.

If your institution or institutions have a press office, please notify them about your upcoming paper to help maximize its impact. If they’ll be preparing press materials, please inform our press team as soon as possible -- no later than 48 hours after receiving the formal acceptance. Your manuscript will remain under strict press embargo until 2 pm Eastern Time on the date of publication. For more information, please contact onepress@plos.org.

Kind regards,

Shivanand Kattimani

Academic Editor

PLOS One

Reviewers' comments:

Reviewer's Responses to Questions

**Comments to the Author**

1. If the authors have adequately addressed your comments raised in a previous round of review and you feel that this manuscript is now acceptable for publication, you may indicate that here to bypass the “Comments to the Author” section, enter your conflict of interest statement in the “Confidential to Editor” section, and submit your "Accept" recommendation.

Reviewer #1: (No Response)

**********

2. Is the manuscript technically sound, and do the data support the conclusions?

The manuscript must describe a technically sound piece of scientific research with data that supports the conclusions. Experiments must have been conducted rigorously, with appropriate controls, replication, and sample sizes. The conclusions must be drawn appropriately based on the data presented.

Reviewer #1: Yes

**********

3. Has the statistical analysis been performed appropriately and rigorously?

Reviewer #1: Yes

**********

4. Have the authors made all data underlying the findings in their manuscript fully available?

The PLOS Data policy requires authors to make all data underlying the findings described in their manuscript fully available without restriction, with rare exception (please refer to the Data Availability Statement in the manuscript PDF file). The data should be provided as part of the manuscript or its supporting information, or deposited to a public repository. For example, in addition to summary statistics, the data points behind means, medians and variance measures should be available. If there are restrictions on publicly sharing data—e.g. participant privacy or use of data from a third party—those must be specified.

Reviewer #1: Yes

**********

5. Is the manuscript presented in an intelligible fashion and written in standard English?

PLOS ONE does not copyedit accepted manuscripts, so the language in submitted articles must be clear, correct, and unambiguous. Any typographical or grammatical errors should be corrected at revision, so please note any specific errors here.

Reviewer #1: No

**********

6. Review Comments to the Author

Please use the space provided to explain your answers to the questions above. You may also include additional comments for the author, including concerns about dual publication, research ethics, or publication ethics. (Please upload your review as an attachment if it exceeds 20,000 characters)

Reviewer #1: I thank the authors for their careful attention to the points raised in the previous round of review. I have verified the revised manuscript against each of the three outstanding items, and I confirm that all three have now been fully and satisfactorily addressed:

1. Interpretive language. The previously flagged expressions (“institutional readiness” in the stated objectives, “persistent mismatch,” “strong attitudinal support,” “overwhelmingly positive,” “broad consensus,” and “favorable environment”) have been replaced throughout the Abstract, Methods, Discussion, and Conclusion with appropriately cautious, exploratory wording (e.g., “generally positive attitudes,” “common agreement among participants,” “potential receptiveness”). The interpretive tone is now well calibrated to a single-center exploratory study with n = 14.

2. Terminology consistency. The correction from “low- and middle-income countries” to “middle-income countries” has now been applied consistently across the entire manuscript, including the Abstract, Introduction, and Discussion. No remaining instances were identified.

3. “Subjective judgment.” All references to subjective judgment, subjective estimation, and impressionistic judgments have been replaced with the neutral term “informal clinical assessment,” used consistently in the Discussion.

My substantive concerns are therefore fully resolved, and I consider the manuscript scientifically ready for acceptance. However, in the course of implementing the revisions, a small number of typographical errors appear to have been introduced (most likely during the acceptance of tracked changes). These do not affect the content but should be corrected before publication:

1. Line 42 (Keywords): a stray letter appears between terms — “Screening; E Perception” should read “Screening; Perception”.

2. Line 102 (Introduction): missing space in “a potentialstep” — should read “a potential step”.

3. Lines 36–37 (Abstract, Conclusion sentence): the comparative construction is incomplete — “challenges … may be related to capacity and organizational support than to resistance” should read “may be related more to capacity and organizational support than to resistance”.

4. Line 477 (Discussion): a double period follows “informal clinical assessments..” — one period should be removed.

5. Line 497 (Discussion): an extra space appears before the period in “organizational support .”

6. Line 500 (Discussion): missing space in “participantsregarding” — should read “participants regarding”.

7. Lines 503–504 (Discussion): the phrase “introducing standardized approaches” is duplicated — “indicates a potential receptiveness to introducing standardized approaches introducing standardized approaches to PPD care” should retain the phrase only once.

8. Line 549 (Abbreviations): “ABREVIATIONS” should read “ABBREVIATIONS”.

Once these minor copyediting corrections are made, I am happy to recommend the manuscript for acceptance. I congratulate the authors on a study that addresses an underrepresented setting in the maternal mental health literature.

**********

7. PLOS authors have the option to publish the peer review history of their article (what does this mean?). If published, this will include your full peer review and any attached files.

If you choose “no”, your identity will remain anonymous but your review may still be made public.

**Do you want your identity to be public for this peer review?**  For information about this choice, including consent withdrawal, please see our Privacy Policy.

Reviewer #1: No

**********

# Editor report: Acceptance letter

**DOI:** 10.1371/journal.pone.0354470.r006

**Authors:** Shivanand Kattimani

PONE-D-26-07800R2

PLOS One

Dear Dr. El Haj Sleiman,

I'm pleased to inform you that your manuscript has been deemed suitable for publication in PLOS One. Congratulations! Your manuscript is now being handed over to our production team.

At this stage, our production department will prepare your paper for publication. This includes ensuring the following:

* All references, tables, and figures are properly cited

* All relevant supporting information is included in the manuscript submission,

* There are no issues that prevent the paper from being properly typeset

You will receive further instructions from the production team, including instructions on how to review your proof when it is ready. Please keep in mind that we are working through a large volume of accepted articles, so please give us a few days to review your paper and let you know the next and final steps.

Lastly, if your institution or institutions have a press office, please let them know about your upcoming paper now to help maximize its impact. If they'll be preparing press materials, please inform our press team within the next 48 hours. Your manuscript will remain under strict press embargo until 2 pm Eastern Time on the date of publication. For more information, please contact onepress@plos.org.

You will receive an invoice from PLOS for your publication fee after your manuscript has reached the completed accept phase. If you receive an email requesting payment before acceptance or for any other service, this may be a phishing scheme. Learn how to identify phishing emails and protect your accounts at https://explore.plos.org/phishing.

If we can help with anything else, please email us at customercare@plos.org.

Thank you for submitting your work to PLOS ONE and supporting open access.

Kind regards,

PLOS ONE Editorial Office Staff

on behalf of

Dr. Shivanand Kattimani

Academic Editor

PLOS One
